# Effectiveness of Telemonitoring for Reducing Exacerbation Occurrence in COPD Patients With Past Exacerbation History: A Systematic Review and Meta-Analysis

**DOI:** 10.3389/fmed.2021.720019

**Published:** 2021-09-10

**Authors:** Jing-wen Lu, Yu Wang, Yue Sun, Qin Zhang, Li-ming Yan, Ying-xi Wang, Jing-han Gao, Yan Yin, Qiu-yue Wang, Xue-lian Li, Gang Hou

**Affiliations:** ^1^Department of Pulmonary and Critical Care Medicine, First Affiliated Hospital of China Medical University, Shenyang, China; ^2^Department of Pulmonary and Critical Care Medicine, Fourth Affiliated Hospital of China Medical University, Shenyang, China; ^3^Department of Epidemiology, School of Public Health, China Medical University, Shenyang, China; ^4^Department of Pulmonary and Critical Care Medicine, Center of Respiratory Medicine, China-Japan Friendship Hospital, Beijing, China; ^5^Department of Pulmonary and Critical Care Medicine, National Center for Respiratory Medicine, Center of Respiratory Medicine, National Clinical Research Center for Respiratory Diseases, Beijing, China; ^6^Institute of Respiratory Medicine, Chinese Academy of Medical Sciences and Peking Union Medical College, Beijing, China; ^7^Department of Pulmonary and Critical Care Medicine, Capital Medical University, Graduate School of Capital Medical University, Beijing, China

**Keywords:** telemonitoring, chronic obstructive pulmonary disease, acute exacerbation of chronic obstructive pulmonary disease, telehealth, telehomecare, telecare, telephone monitoring, telemedicine

## Abstract

**Background:** Although an increasing number of studies have reported that telemonitoring (TM) in patients with chronic obstructive pulmonary disease (COPD) can be useful and efficacious for hospitalizations and quality of life, its actual utility in detecting and managing acute exacerbation of COPD (AECOPD) is less established. This meta-analysis aimed to identify the best available evidence on the effectiveness of TM targeting the early and optimized management of AECOPD in patients with a history of past AECOPD compared with a control group without TM intervention.

**Methods:** We systematically searched PubMed, Embase, and the Cochrane Library for randomized controlled trials published from 1990 to May 2020. Primary endpoints included emergency room visits and exacerbation-related readmissions. *P*-values, risk ratios, odds ratios, and mean differences with 95% confidence intervals were calculated.

**Results:** Of 505 identified citations, 17 original articles with both TM intervention and a control group were selected for the final analysis (*N* = 3,001 participants). TM was found to reduce emergency room visits [mean difference (MD) −0.70, 95% confidence interval (CI) −1.36 to −0.03], exacerbation-related readmissions (risk ratio 0.74, 95% CI 0.60–0.92), exacerbation-related hospital days (MD −0.60, 95% CI −1.06 to −0.13), mortality (odds ratio 0.71, 95% CI 0.54–0.93), and the St. George's Respiratory Questionnaire (SGRQ) score (MD −3.72, 95% CI −7.18 to −0.26) but did not make a difference with respect to all-cause readmissions, the rate of exacerbation-related readmissions, all-cause hospital days, time to first hospital readmission, anxiety and depression, and exercise capacity. Furthermore, the subgroup analysis by observation period showed that longer TM (≥12 months) was more effective in reducing readmissions.

**Conclusions:** TM can reduce emergency room visits and exacerbation-related readmissions, as well as acute exacerbation (AE)-related hospital days, mortality, and the SGRQ score. The implementation of TM intervention is thus a potential protective therapeutic strategy that could facilitate the long-term management of AECOPD.

**Systematic Review Registration:** This systematic review and meta-analysis is reported in accordance with the Preferred Reporting Items for Systematic Reviews and Meta-Analyses (PRISMA) Statement and was registered at International Prospective Register of Systematic Reviews (number: CRD42020181459).

## Introduction

Chronic obstructive pulmonary disease (COPD) refers to a progressive, irreversible disease characterized by persistent airway limitation, and its symptoms often worsen over time (1, 2). Acute exacerbation of COPD (AECOPD) is defined as an acute worsening of respiratory symptoms affecting patients' health status, lung function, and COPD-related costs (2, 3). Acute exacerbations (AEs) account for ~70% of COPD-related direct medical costs, with over 18 billion spent on direct costs annually worldwide (4). Given the magnitude of these numbers, using telehealth to achieve even a small percentage gain in savings is of interest (5). Despite the substantial impact that exacerbations may have, patients with COPD often have difficulty recognizing early deterioration based on symptoms and cannot respond adequately or in a timely manner, indicating an urgent need to develop effective management options to help patients recognize the early onset of AECOPD (6, 7).

Telemonitoring (TM) refers to the use of electronic information and communication technologies to support distance healthcare, allowing information exchange between supervising clinicians and long-distance patients regarding symptoms or physiological measurements and enabling access to healthcare services (5, 8, 9). TM has been using a wide range of technological devices, varying from an information and communication technologies platform including a web-based call center (10); a tablet cable computer with a web camera, a microphone, and measurement equipment (11); electronic diary on the website (12); a telephone line to a central data management unit to a monitoring platform *via* a touch-screen computer and a mobile modem (13), etc. TM has recently begun to be used for the management of patients with COPD (14, 15). It has attracted interest as a potential solution to the global challenge of providing care for aging populations and thus may also be an alternative to self-management (SF) to reduce the impact of AECOPD (6, 16, 17). The early detection of exacerbations may decrease healthcare costs by informing individualized interventions to prevent further exacerbation events, decelerate disease progression, and reduce mortality (18). Since 2006, there have been a lot of studies on TM for remote periodic management of COPD (10). Recent studies have reported that TM may be beneficial for COPD patients (14, 19–22), whereas others have shown that TM is unlikely to result in statistically significant improvements in the exacerbation rate (23, 24), so there were no consistent conclusions that have been reached on whether it can reduce exacerbations. As previous studies revealed, a patient with past exacerbation histories would have more risks for future exacerbations (25), and the reduction of exacerbations is one of the current COPD management goals gaining enthusiastic promotion by policy makers (26). It was supposed that the inclusion criteria of various studies with or without past exacerbation histories might produce different conclusions. Therefore, as for the goal of reducing the frequent exacerbations, we designed to conduct this meta-analysis focusing on patients with exacerbations in the past 12 months with the potential significance who might benefit from TM so as to provide an answer for the appropriate selection of TM for COPD patients.

## Materials and Methods

### Search Strategy and Selection Criteria

Three English databases, PubMed, Embase, and the Cochrane Central Register of Controlled Trials (CENTRAL), were comprehensively searched. The language was limited to English only, with no date restrictions, allowing retrieval of papers from the inception of the databases to May 2020. Both keywords and Medical Subject Heading (MeSH) terms were used, including “Pulmonary Disease, Chronic Obstructive,” “telemonitoring,” “telecare,” “telehomecare,” “telehealth,” “telephone monitoring,” “telemedicine,” “telepathology,” “telecommunication,” “Disease Progression,” “exacerbation,” and combinations of these search terms. The reference lists of all the included studies were examined for relevant articles from 1990 to May 2020. The search strategy used in this study is included in the Supplementary Material.

### Study Selection

The inclusion criteria were as follows: (a) randomized controlled trials (RCTs), including pilot studies; (b) patient diagnosed with COPD according to the Global Initiative for Chronic Obstructive Lung Disease (GOLD): had a post-bronchodilator forced expiratory volume in 1 s (FEV_1_)/forced vital capacity <0.70; (c) a TM intervention (telemedicine, telehealthcare, telerehabilitation, teleconsultation, telecare, telehealth, mobile tool, apps, call center, etc.); (d) the TM device should periodically monitor significant parameters or symptoms and transmit these records to the researchers; (e) comparison with a control group (usual care, ordinary health care, blank control, face-to-face support, etc.); (f) reporting at least one of the following exacerbation-related main outcomes: emergency room (ER) visits, readmissions, or mortality; (g) patients had at least one exacerbation or hospitalization/ER visit due to COPD in the past 36 months; and (h) the observation period was at least 6 months.

The exclusion criteria were as follows: (a) duplicate study; (b) nonrandomized controlled trial; (c) not an original article (e.g., review papers, editorials, commentaries on articles, study protocols, abstracts of communications or meetings review articles, conference posters, and unpublished gray literature); (d) comparison between two different TM interventions; (e) included only regular telephone calls, video consultation, or teleconference interventions without clinical TM data; and (f) not published or translated in English.

### Data Extraction and Quality Assessment

The data extraction strategy is provided in the Supplementary Materials. The extracted data included first author, year of publication, region, study design, duration of study, sample size, age, sex, lung function, characteristics of recruited patients with COPD, characteristics of the intervention, control group, study outcomes, and results.

The risk of bias of the included studies was evaluated by two reviewers (Y.W. and Y.S.) according to the Cochrane Collaboration risk of bias tool for RCTs, as shown in the Supplementary Material.

### Outcome Measures

#### Primary Endpoints

In this meta-analysis, the primary endpoints were moderate to severe exacerbations, defined as those resulting in a visit to the ER or hospital admission (9). Hence, ER visits or readmissions are used as proxies for the exacerbation rate. We classified the admission into AE-related and all-cause admissions since the management and follow-ups of admissions could be tracked with the outcomes and causes of admissions. However, for the ER visits, most studies {except only one (27)} did not present the definite cause classifications of ER visits. We supposed that ER visits had various outcomes, such as admission, discharge from ER, transfer to other medical centers, etc. Thus, it was difficult to track the outcomes and causes of admissions. So, we did not divide ER visits into AE-related and all-cause ER visits. The outcomes reported in this article were estimated at the longest follow-up. For the study purpose, we considered the following outcomes as the primary endpoints (Table 1):

1. ER visits;
2. Readmissions: exacerbation-related (AE-related) readmissions, all-cause readmissions, and the rate of AE-related readmissions.

**Table 1:** TM studies and pooled clinical outcomes.

**Clinical outcomes**		**Type of variable**	**No. of studies**	**No. of patients**
**Primary endpoints**				
ER visits		Continuous	6 (5, 11, 12, 27–29)	1,099
Re-admissions	AE-related readmissions	Dichotomous	7 (10–13, 17, 30, 31)	1,281
	Rate of AE-related readmissions	Continuous	9 (5, 6, 9–12, 14, 28, 31)	1,573
	All-cause readmissions	Dichotomous	4 (10, 11, 16, 17)	772
**Secondary endpoints**				
LOS	AE-related hospital days	Continuous	6 (5, 11, 13, 14, 17, 28)	1,073
	All-cause hospital days	Continuous	7 (9, 11, 13, 14, 16, 29, 31)	2,201
Mortality		Dichotomous	11 (5, 9–11, 13, 14, 28, 29, 31–33)	2,307
Time to first hospital readmission		Continuous	3 (14, 16, 31)	887
HRQoL	SGRQ, total	Continuous	6 (14, 27–29, 31, 33)	1,212
	EQ-5D, change	Continuous	2 (6, 28)	195
	EQ-VAS, change	Continuous	2 (6, 28)	195
Anxiety and depression	HADS-A, change	Continuous	2 (13, 28)	444
	HADS-D, change	Continuous	2 (13, 28)	444
Physical capacity	6MWT, distance, change	Continuous	2 (30, 32)	81

*Readmission is defined as hospitalization to the same or different hospital for any reason within the following year after discharge (9). LOS was defined as hospital days per admission (days), and the number of days of an admission was calculated as the number of midnights during the admission dates, with the exception of a patient discharged on the same day who was allocated a 1-day LOS (5). TM, telemonitoring; ER, emergency room; AE, acute exacerbation; LOS, length of stay; HRQoL, health-related quality of life; SGRQ, St. George's Respiratory Questionnaire; EQ-5D, EuroQol five-dimension scale; EQ-VAS, EuroQol visual analog scale; HADS-A, Hospital Anxiety and Depression Scale-Anxiety; HADS-D, Hospital Anxiety and Depression Scale-Depression; 6MWT, 6-min walking test*.

#### Secondary Endpoints

For the study purpose, we considered the following outcomes as the secondary endpoints:

1. Length of stay (LOS): AE-related hospital days and all-cause hospital days;
2. Mortality;
3. Time to first hospital readmission;
4. Health-related quality of life (HRQoL): St. George's Respiratory Questionnaire (SGRQ, total score) and change in the EuroQol five-dimension scale (EQ-5D) and EuroQol visual analog scale (EQ-VAS) scores between baseline and the end of the study;
5. Anxiety and depression: change in Hospital Anxiety and Depression Scale (HADS-A and HADS-D) scores between baseline and the end of the study;
6. Exercise capacity: change in the 6-min walking test (6MWT) distance between baseline and the end of the study.

### Data Synthesis and Analysis

A narrative description of each study was produced. The data from the RCTs were analyzed using intention-to-treat protocols. The variable analysis and data synthesis methods are shown in the Supplementary Material.

For the outcomes of interest, prespecified subgroup analyses were performed based on the duration of follow-up, as this factor may affect the impact of the intervention. Shorter-term (no more than 6 months) and longer-term (more than 6 months) effects of TM interventions may differ. In addition, we performed exploitative analyses by using different cutoff points for follow-up times (e.g., 6, 9, and 12 months).

In addition, a sensitivity analysis was carried out to examine the stability of the combined results for the primary outcomes only under different assumptions and thereby investigate the robustness of the effect sizes found in this review. We were unable to use statistical methods (e.g., funnel plots and Egger's regression test) to assess publication bias because the number of studies included in the analysis was small (*n* < 10). Hence, sensitivity analyses were performed to identify whether the review findings were dependent on study characteristics using random-effects vs. fixed-effects modeling or by analyzing specific populations (patient number ≥100 or TM with SF intervention). We conducted all meta-analyses with Review Manager, version 5.3.

## Results

### Characteristics of the Included Studies

**Figure 1:**
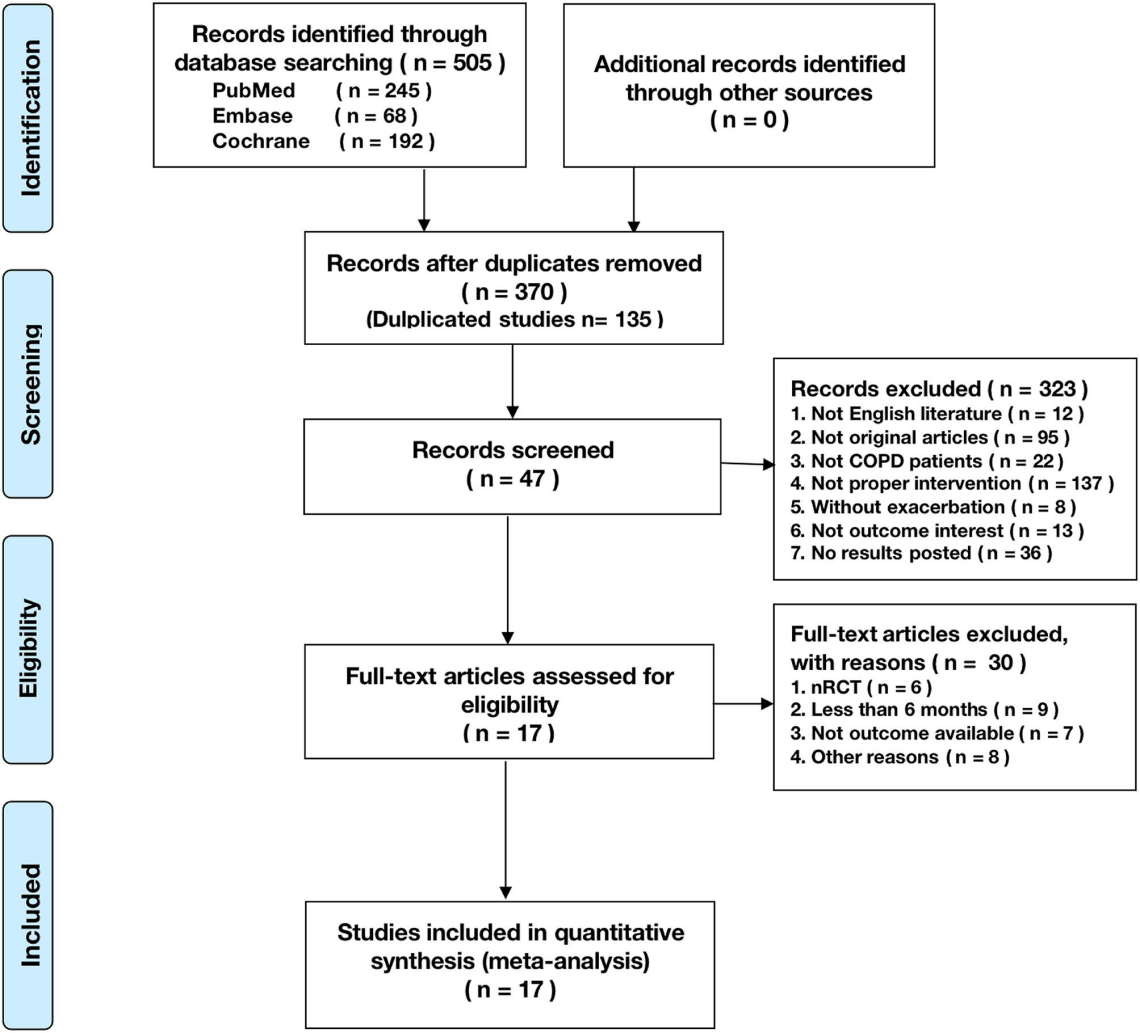
The Preferred Reporting Items for Systematic Reviews and Meta-Analyses (PRISMA) flowchart reporting the number of papers identified, screened, and excluded.

**Figure 2:**
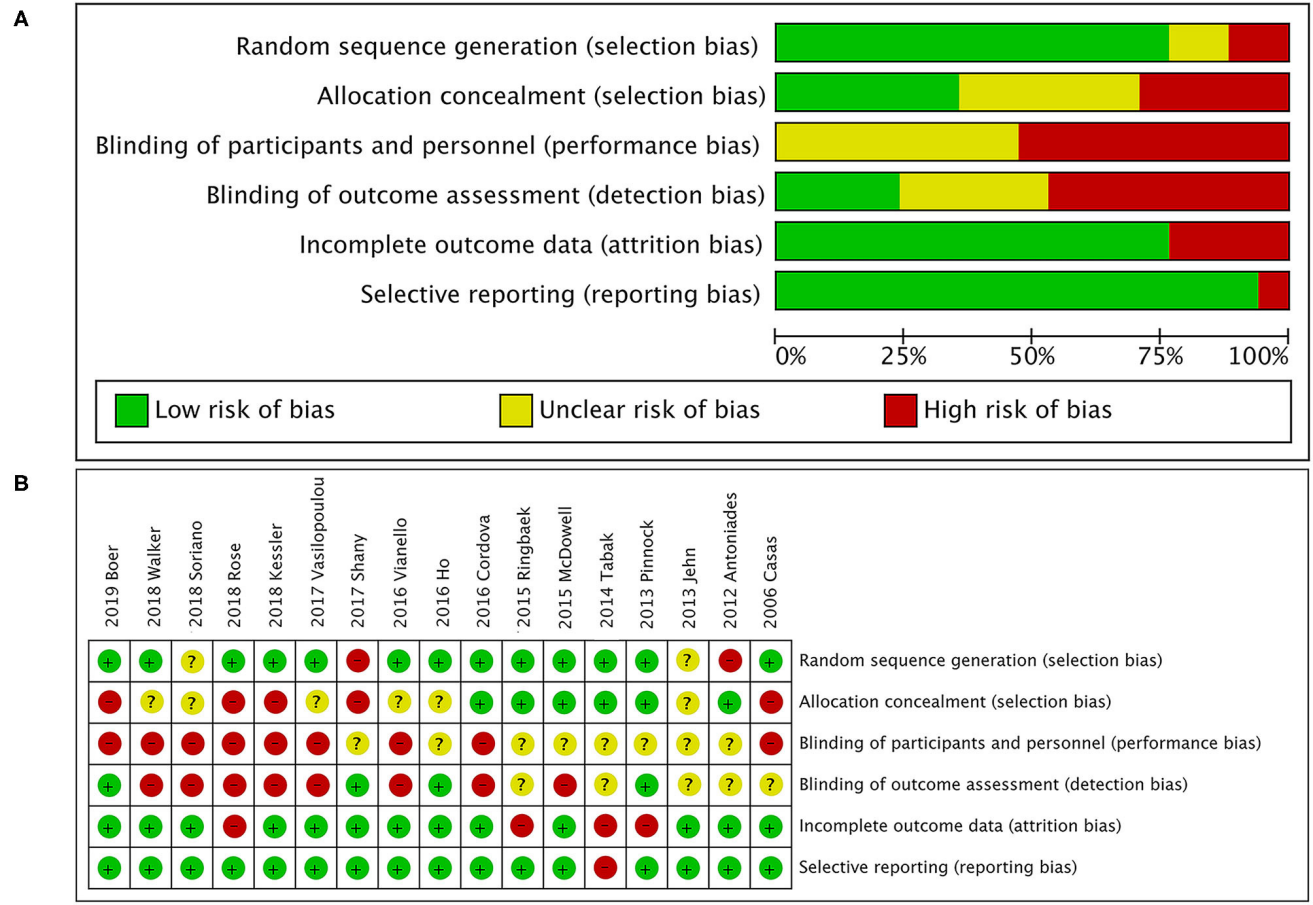
Quality assessment. **(A)** Risk of bias graph; **(B)** risk of bias summary. The overall risk of bias in randomized controlled trials (RCTs) was rated as moderate to high for issues related to blinding. Owing to the type of intervention, patients and health care research team could not be blinded for group assignment, as it was responsible for the personalized and technical support of the TM tool. In addition, most studies had reasonable random sequence generation. However, only six studies specified whether data collectors and outcome assessors were masked to treatment allocation.

A total of 505 citations were identified by a comprehensive search of the literature; 17 articles involving 3,001 people were identified as relevant to this study, and these publications were ultimately selected for inclusion in the meta-analysis for critical appraisal (Figure 1; Supplementary Table 1) (5, 6, 9–14, 16, 17, 27–33). Thirty studies were excluded after evaluation for the reasons documented in Figure 1. The assessment of patients before enrollment in the study was identical for both groups in terms of (1) the SGRQ total score and (2) history of previous exacerbations requiring inpatient hospitalizations/ER visits. The participants in the intervention group and control group received the same clinical care and had access to the same healthcare services. The only difference between the two groups was that the former received TM services. Six studies showed beneficial effects of TM intervention on COPD-related clinical outcomes (5, 10, 12, 17, 30, 33), and 11 studies showed that it did not reduce exacerbations (6, 9, 11, 13, 14, 16, 27–29, 31, 32). Figure 2 shows the assessment of risk of bias in the trials. Various remote TM of vital signs allows clinicians to monitor a COPD patient remotely with reference and availability of the physiological signs, respiratory symptoms, and activity levels in a diffuse manner due to the technology updating. In order to make the diffusiveness clearly presented, we added a table as the summary of the TM methods and comparison in supplement (Supplementary Table 3).

### ER Visits

**Figure 3:**
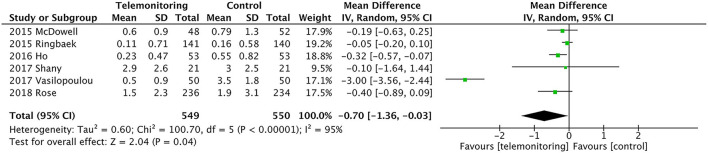
A meta-analysis of ER visits. Df, degrees of freedom; M-H, Mantel–Haenszel.

Six RCTs provided data comparing ER visits between the TM and control groups (5, 11, 12, 27–29). The TM group had fewer ER visits [*P* = 0.04, mean difference (MD) = −0.70, 95% confidence interval (CI) (−1.36, −0.03)]. High heterogeneity was found (*P* = 0.000, *I*^2^ = 95%). When we reviewed the included studies again because of the substantial degree of heterogeneity, we found one study by Vasilopoulou et al. in which ER visits due to AECOPD but not associated with hospital admission might reflect a different degree of AECOPD severity than in other studies and had more frequent data transmitting interval for 5 days per week, 10 h per day, which made the patients have excellent adherence and very good compliance (27). When we performed sensitivity analysis by removing this study from the analysis, the heterogeneity dropped from *I*^2^ = 94% to *I*^2^ = 9%. An analysis of the total population showed significantly fewer ER visits in the TM group [*P* = 0.02, MD = −0.14, 95% CI (−0.26, −0.02), *I*^2^ = 9%]; see Figure 3.

We omitted the studies with relatively small sample sizes (*n* < 100) or TM without SF to perform a sensitivity analysis and to examine the stability of the pooled results for ER visits. As shown in Table 2, no significant effect was observed from the exclusion of any single study, and the pooled results indicated good stability (MD = −0.76, *P* = 0.04). There were also no differences in the results between the fixed and random statistical effects (MD = −0.27; *P* = 0.000).

**Table 2:** Sensitivity analyses specific to sample size ≥100 or intervention of TM with SF.

**Methods**	**Main endpoints**	**No. of studies**	**No. of patients**	**MD/RR**	**95% CI**	***P***	***I* ^**2**^**
Sample size ≥100	ER visits	5 (11, 12, 27–29)	1,057	MD = −0.76	[−1.47 to −0.05]	0.04	96%
	AE-related readmissions	5 (10–13, 31)	1,195	RR = 0.82	[0.69, 0.97]	0.02	66%
TM with SF intervention	ER visits	3 (27–29)	670	MD = −0.98	[−1.26 to −0.69]	0.00	97%
	AE-related readmissions	4 (10, 13, 17, 31)	591	RR = 0.87	[0.79, 0.94]	0.01	26%

*The effect of the pooled estimates for the studies in which the sample size was over 100 or the intervention was TM plus SF did not significantly alter the effect on ER visits and AE-related readmissions. The power of studies with small sample size (<100) (5, 17, 30) did not meet the minimum requirements of the research (0.34, 0.37, and 0.66, respectively). So, after the exclusion of the above studies with small sample size, the conclusion was in accordance to the overall conclusion. Similarly, the alike pattern was found in TM-plus-SF populations. In other words, after the sensitivity analysis, it was proved that our final conclusion was consistent with the conclusion from the subgroup analysis of specific populations (patient number ≥100 or TM-plus-SF intervention group). MD, mean difference; RR, risk ratio; CI, confidence interval*.

### Readmission

#### AE-Related Readmissions

**Figure 4:**
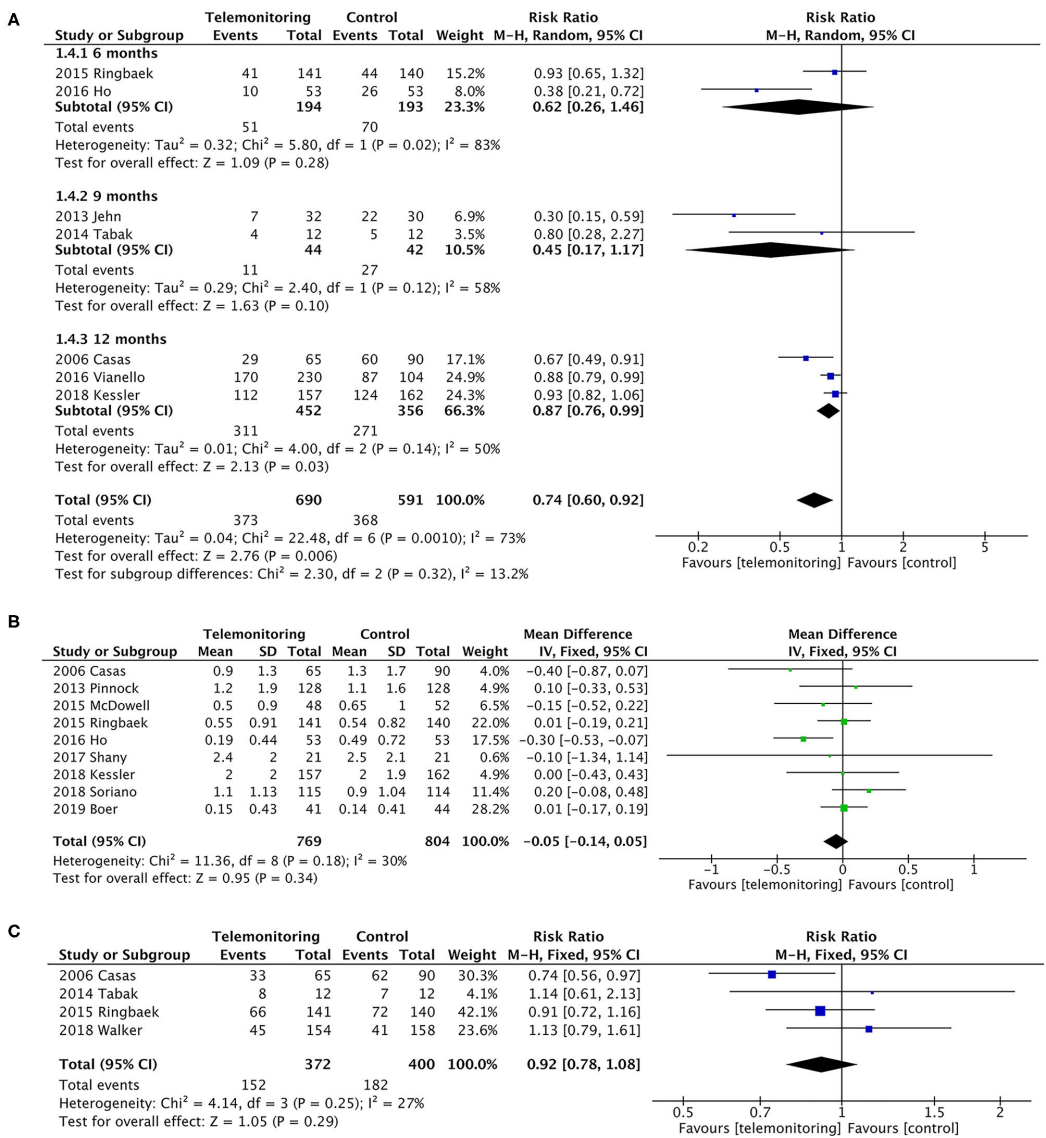
A meta-analysis of readmissions. **(A)** Exacerbation-related readmissions (subgroup analysis with observation period). A sensitivity analysis using the fixed-effect model resulted in a similar effect size [RR = 0.81, 95% CI (0.74–0.88)] compared to random-effects modeling. One study (Soriano et al.) (9) could not be included in the meta-analysis for it only collected the number of participants who have at least one exacerbation (ER visit or hospitalization) in the past 12 months, resulting in the failure of exacting the required data. Subgroup analysis. In the subgroup in which follow-up duration was 6 or 9 months, it did not reduce readmissions [*P* = 0.28, RR = 0.62, 95% CI (0.26, 1.46) and *P* = 0.10, RR = 0.45, 95% CI (0.17, 1.17), respectively]. Statistically, heterogeneity was found in both subgroups (*I*^2^ = 83% and 58%). However, the subgroup of 12 months showed a greater reduction on readmissions [*P* = 0.03, RR = 0.87, 95% CI (0.76, 0.99), *I*^2^ = 50%]. Additionally, a sensitivity analysis was performed for the primary outcome to test an overall pooled effect. The results were no different between fixed and random statistical effects (RR = 0.81; *P* = 0.000). **(B)** The rate of exacerbation-related readmissions. **(C)** All-cause readmissions. Low heterogeneity was found (*P* = 0.29, *I*^2^ = 27%).

In a pooled analysis of all seven RCTs (10–13, 17, 30, 31), the usage of TM led to a greater reduction in exacerbation-related readmissions than the control treatment, with statistically significant between-study heterogeneity [*P* = 0.006, risk ratio (RR) = 0.74, 95% CI (0.60, 0.92), *I*^2^ = 73%]. Then, the results were stratified by the observation period, and the subgroup analysis compared the periods of 6 or 9 months with that of 12 months. The effect of sequentially recalculating the pooled estimates for the studies in which the sample size was over 100 or the intervention was TM plus SF did not significantly alter the effect on AE-related readmission (RR = 0.82, *P* = 0.02; RR = 0.87, *P* = 0.01, respectively). Details are shown in Figure 4; Table 2.

#### Rate of AE-Related Readmissions

Calculations were performed using a continuous variable for the rate of hospitalizations for each patient. The pooled results of the nine trials for the rate of exacerbation-related readmissions were homogeneous (*P* = 0.34), and the mean difference was −0.05 (95% CI was −0.14–0.05) in favor of TM (5, 6, 9–12, 14, 28, 31). A slight heterogeneity was found (*P* = 0.18, *I*^2^ = 30%). Only four of the nine trials, however, showed a benefit of TM (Figure 4) (5, 10, 12, 28).

#### All-Cause Readmissions

The dichotomous variables included the number of readmissions for any cause. Four studies were included (10, 11, 16, 17), with no statistically significant difference [*P* = 0.29, RR = 0.92, 95% CI (0.78, 1.08)]; see Figure 4.

### Secondary Outcomes

**Figure 5:**
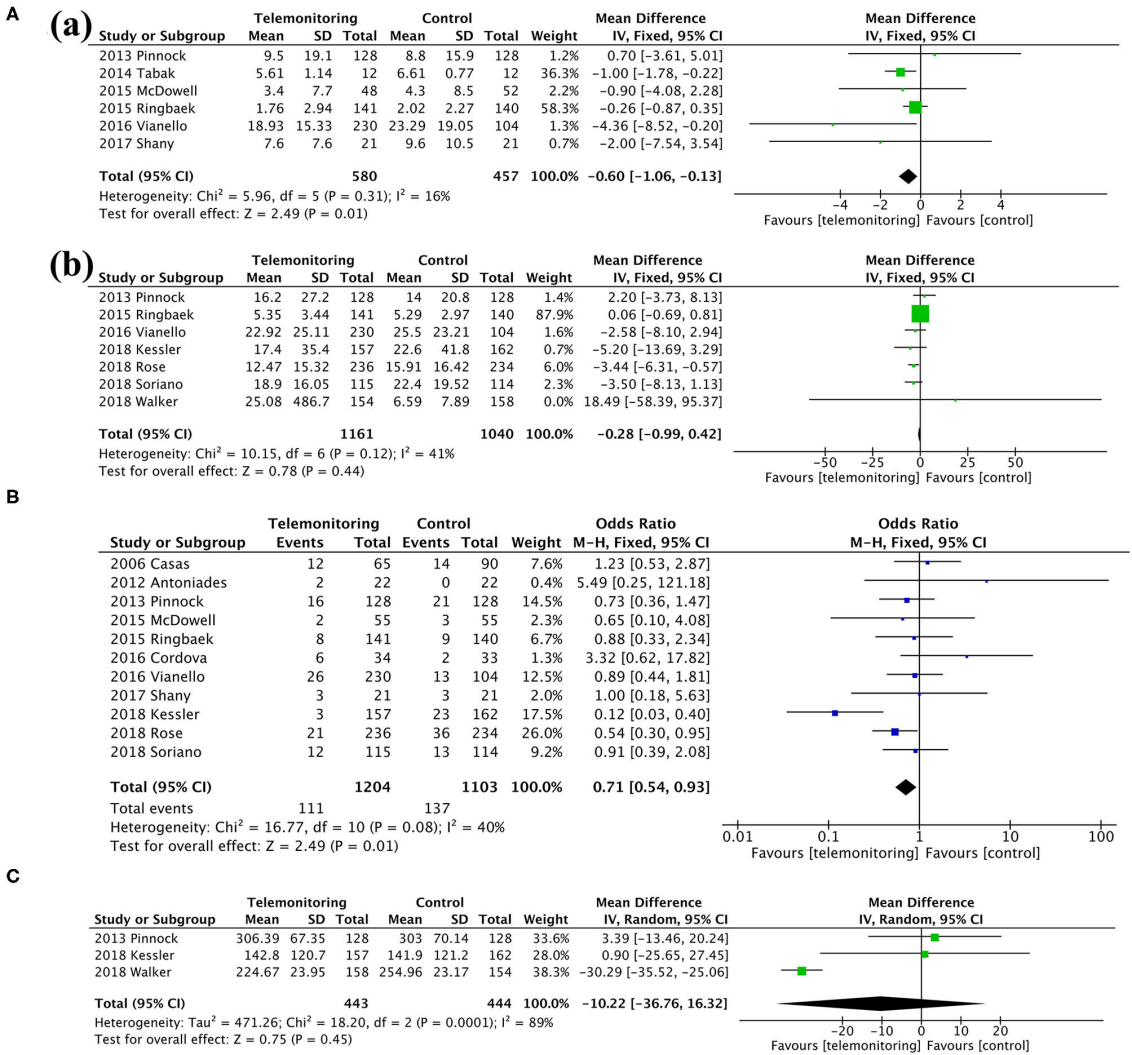
A meta-analysis of secondary outcomes. **(A)** Length of stay: (a) exacerbation-related hospital days and (b) all-cause hospital days. **(B)** Mortality. **(C)** Time to first hospital readmission.

**Figure 6:**
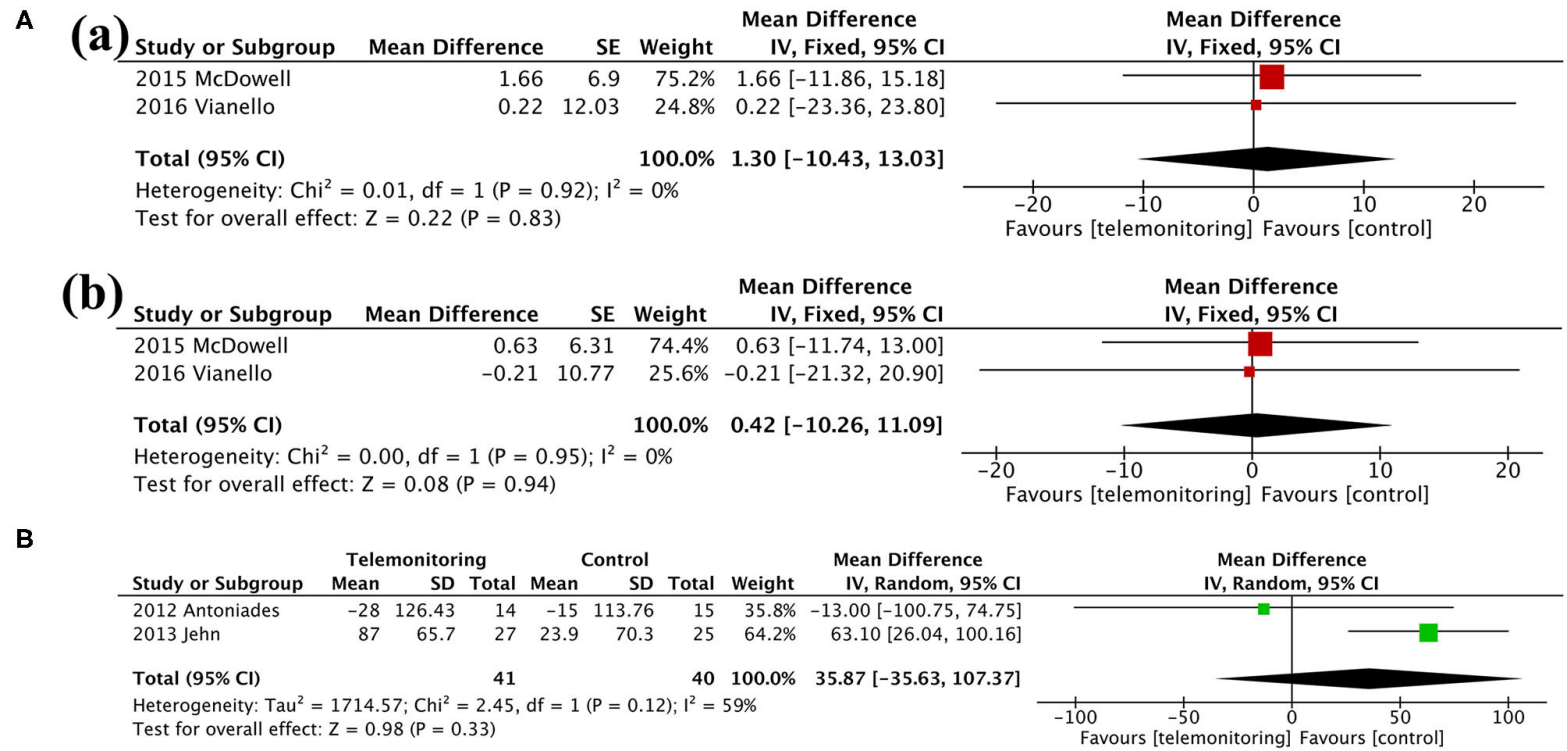
Meta-analysis. **(A)** Anxiety and Depression: (a) HADS-A and (b) HADS-D. **(B)** Exercise capacity: 6MWT. HADS-A, Hospital Anxiety and Depression Scale-Anxiety; HADS-D, Hospital Anxiety and Depression Scale-Depression; 6MWT, 6-min walking test.

Among the secondary outcomes observed, AE-related hospital days, mortality, and the SGRQ score, representing quality of life, were improved by TM [AE-related hospital days, MD = −0.60, 95% CI (−1.06, −0.13), *P* = 0.01; mortality, OR = 0.71, 95% CI (0.54, 0.93), *P* = 0.01; SGRQ score, MD = −3.72, 95% CI (−7.18, −0.26), *P* = 0.04], as shown in Figures 5, 6; Table 3. The detailed results regarding the secondary outcomes are shown in the Supplementary Materials.

**Table 3:** The pooled results of HRQoL of TM on AECOPD.

**Clinical outcomes**	**No. of studies**	**No. of patients**	**Pooled results**			
			***P***	**MD**	**95% CI**	***I* ^**2**^**
SGRQ (total), baseline	10 (5, 10, 14, 16, 27–31, 33)	1,893	0.44	−0.65	[−2.27, 0.98]	0%
SGRQ (total), outcome	6 (14, 27–29, 31, 33)	1,212	0.04	−3.72	[−7.18 to −0.26]	54%
EQ-5D, change	2 (6, 28)	195	0.9	−0.03	[−0.45, 0.40]	0%
EQ-VAS, change	2 (6, 28)	195	0.84	4.54	[−39.37, 48.45]	0%

## Discussion

Patients with COPD, most of whom are elderly, often have difficulty recognizing early symptom deterioration and do not respond in a timely or adequate manner in the course of symptom worsening (27). TM technology is constantly being improved and has developed from monitoring daily parameters transmitted to a call center *via* computers to simply tapping a touch screen or app on a mobile phone, complemented by measurements with a pulse oximeter, spirometer, and other instruments (6, 32). To date, studies on the effects of electronic healthcare in the management of chronic disease have focused on the use of applications of and wearable devices (6, 34). Remote monitoring of vital signs enables clinicians to track a patient's physical signs and respiratory symptoms from a distance using a wide range of technical equipment for the early detection of exacerbations (27). Telehealth technology has become more convenient and could promote patient–physician communication, facilitating patients' ability to promote increased levels of physical activity and health status and increasing their awareness of compliance with treatment (17). In this context, telehealthcare has been proposed as a novel management strategy that could minimize the likelihood of exacerbation and hospitalization. However, the results of the effect of TM on AECOPD are still uncertain, especially in the population with high risks of AECOPD. The findings of our meta-analysis provide evidence that TM reduces exacerbation-related rehospitalizations and ER visits in the long term for patients with a past AECOPD history. In our analysis, all studies were RCTs; however, some of them had relatively small sample sizes (*n* < 50) (5, 17, 32), and most of the studies were unblinded {except four articles that were researcher blinded (5, 6, 12, 14)} due to the necessity of the monitoring equipment. The principal findings of this systematic review imply that TM reduced ER visits, AE-related readmissions, AE-related hospital days, mortality, and the SGRQ score but did not make a difference in the rate of AE-related readmissions, all-cause readmissions, all-cause hospital days, time to first hospital readmission, change in the EQ-5D score, change in the EQ-VAS score, anxiety and depression, or exercise capacity. As a consequence, the result was in favor of the usage of TM as a protective strategy in the management of AECOPD. Additionally, after a sensitivity analysis including studies with a larger sample size or a TM-plus-SF intervention, similar results were found.

The strongest predictor of the frequency of future exacerbation remains the number of exacerbations in the prior year (35, 36). Patients hospitalized for COPD exacerbation are at higher risk of readmission in the following year; hence, we aimed to reduce these adverse events in patient care (12). Thus, we only included studies that had a population of high-risk patients with a history of exacerbations, as the majority of them experienced ER visits or hospitalizations before enrollment in the trials. Exacerbations become more frequent as COPD progress (37). Regarding the AE-related readmission rate and the mean number of AE-related readmissions, we had controversial results, which can at least in part be explained by the fact that the meaningful index for the frequent AECOPD patients was the occurrence of AE but not the frequency of AE. In other words, the history of AECOPD but not the mean number of AECOPD indicates high risks of exacerbations. Similarly, the studies containing both outcomes had consistent results that TM used to care for patients with COPD exacerbation improves outcomes in terms of COPD-related readmissions, but not in average number of AE-related readmissions (11, 31).

In regard to the comparison between the beneficial effects on exacerbation-specific readmissions and all-cause readmissions, a better detection sensitivity of TM for AE-related admissions was found but not in all-cause readmissions. This can be explained by the fact that the home telemedicine group had the advantage of spirometry and physical symptom monitoring (markers of reliable predictors of AECOPD), which may have more rapid detection of respiratory symptoms or deterioration of lung function leading to timely medical treatment. Unsurprisingly, it is reasonable to assume that these physiological parameters cannot always reflect changes in patients' health status, which may reduce the ability to recognize a wide range of comorbidities (e.g., heart failure, diabetes, cancer, etc.), resulting in non-significant difference in all-cause readmissions. Consequently, our results were in accordance with most previous studies (20–22). Our exacerbation-related secondary outcomes, similarly, demonstrated the same pattern for AE-related hospital days and all-cause hospital days.

Findings from our meta-analysis showed an overall beneficial effect on the SGRQ score over 6 months. However, the total SGRQ score results were inconsistent in other articles (20, 22, 24, 38). This may be due to the different parameters monitored. Monitoring respiratory parameters (e.g., SpO_2_ and HR) alone might be an optimal choice for ensuring beneficial effects on respiratory-related quality of life. In addition, the lack of improvement in the EQ-5D score, which describes and evaluates the health status of patients in broad disease areas, could be explained by the limited and specific monitoring range of respiratory system. In line with the lack of evidence of an improved EQ-5D score, TM did not seem to have a positive impact on patients' emotional distress, in particular on the severity of anxiety and/or depression (13, 28). Previous studies showed that anxiety and depression are related to the oxygen saturation, breathlessness, or activity endurance of patients but not to acute exacerbation frequency (39). Even though TM devices could provoke anxiety under the SF approach, we found no differences between the two groups regarding psychiatric disease. Therefore, early detection of AE through TM could not ameliorate psychological problems. Furthermore, the lack of change in the 6MWT distance suggested that the intervention could not improve exercise capacity either (30, 32).

In our meta-analysis, significant diffuseness of TM could be observed. TM technology has constantly developed and reformed, from monitoring of daily parameters transmitted to a call center *via* computers, simply tapping on the touch screen, to applications on the mobile phone complemented by measurements with a pulse oximeter and spirometer. In a word, TM technology developed with the update of technology of information, communication, computer, etc. As time went by, the relatively old-fashioned remote TM was updated and replaced by the newly developed technologies. Thus, in this meta-analysis, the interventions of TM presented as inevitable and wide-ranged diffuseness. For the effect of some specific type of TM, due to the rapid progress and update of technology, studies were quite limited for the conclusion for one specific TM device. However, for the overall effect of the TM intervention, the evidence was sufficient and the conclusion was robust. To optimize engagement, TM interventions would be attractive, rewarding, safe, tailored to patient needs, adapt seamlessly to variations in local connectivity, as well as provide flexibility in monitoring capability to meet individual clinical need. Nonetheless, it would still be challenging for the relatively high economic cost of telehealth for chronic disease due to the long-time usage, which restricts its implementation in the majority of healthcare settings. Thus, more researches are being needed on the clinical effects of the TM tools when used appropriately.

### Strengths and Limitations

An advantage of our study was that we aimed to determine the long-term clinical effectiveness of TM on exacerbations. The studies included in our review had a median follow-up of 12 months, and the minimum was 6 months. Our study analyzed subgroups by observation period. When the duration was 12 months, TM reduced rehospitalization to a greater extent than at 6 or 9 months, which did not significantly reduce readmission.

Additionally, some studies discussed patient compliance and satisfaction rates (16, 17, 32). Though the lack of data availability from some studies did now allow pooling of the data, all these studies drew consistent conclusions that patients were very positive about the benefits of TM.

The deployment of TM for AECOPD had a favorable effect on ER visits, readmissions, quality of life, and cost-effectiveness based on a long-term perspective, especially an observation period of more than 12 months. Nonetheless, more detailed research is needed to fully understand its potential. Additionally, we studied TM in a special group of patients with COPD who had a previous episode of exacerbation requiring hospitalization, indicating the high risks of exacerbations, and confirmed the validity of TM. Thus, the application of TM for COPD may provide the potential ability for the early detection of AECOPD and the initiation of the early and timely management of disease.

Our review has several limitations. First, in RCTs performed in the COPD population included in our analysis, for controlled comparison, spirometry criteria in the GOLD guideline is employed as the inclusion criteria. Surely, due to many patients without spirometry are clinically diagnosed with COPD, we would miss many individuals treated for COPD, and studies in the real world might serve as an important complement to RCTs. Secondly, although most of the included studies were RCTs, a potential risk of bias was found in several domains and lack of some information, such as with respect to a lack of blinding and selection bias, which also reduced the possibility of drawing robust conclusions. In fact, it is not clear by what mechanisms telemonitoring works. More studies are required to answer the way patients are changed and managed (e.g., more attentive medical team, better knowledge of the patients and their abilities to follow instructions, better compliance, etc.). Besides, we conducted a sensitivity analysis to investigate differences in effect size and in strength of conclusions. If the sufficient power was not satisfied (generally set as 0.8), the results obtained were not credible and influence the final conclusion. Thus, we performed the sensitivity analysis. Based on the current sample size (<100), the power of the excluded studies (5, 17, 30) calculated were 0.34, 0.37, and 0.66, respectively, which did not meet the minimum requirements of the research. After the exclusion of the above studies with small sample size, the conclusion was in accordance to the overall conclusion. In other words, after the sensitivity analysis, it was proved that our conclusion was consistent with the conclusion from the subgroup analysis of specific populations (patient number ≥100) and sufficient power was satisfied. However, after the exclusion of the studies with small sample size, there might be publication bias. Third, even though TM cases and controls were given the same basic instructions that differed only regarding the TM intervention, the complexity of the basic instructions, especially the high quality of usual care, might make the results less accurate or underestimate the effectiveness of TM due to the good control of disease, which reduces the AECOPD. Fourth, with the rapid development and update of technologies, wide-ranged diffuseness was inevitable. Finally, issues such as patient adherence, satisfaction rate, and cost were not established due to the lack of published information. A cost-effectiveness analysis was based on the cost of the intervention during the study period. We could not synthesize this outcome in a meta-analysis because the expenditure of each patient differed across countries and devices. Moreover, the cost difference varied between patients, being greatest in those who were hospitalized in the previous year.

## Conclusions

Based on the available evidence presented in this meta-analysis, TM actually reduced ER visits and AE-related readmissions and reduced AE-related hospital days and mortality in patients with AECOPD, especially when the TM intervention was carried out for more than 12 months. The rapid progress and reformation of TM in practice might require more repeated control studies to conclude the effect and benefit of some special TM types. Thus, TM represents a new option for the management of the disease.

## Data Availability Statement

The original contributions presented in the study are included in the article/Supplementary Material, further inquiries can be directed to the corresponding author/s.

## Author Contributions

GH determined the conception and design of the work, searched the literature, selected targeting studies, extracted and interpreted the data, conducted the meta-analysis, wrote the manuscript, and approved the final version of the manuscript. GH and J-wL carried out interpretation of data for the work. J-wL, YW, and YS assessed risk of bias and grade of the evidence. YW, YS, QZ, L-mY, Y-xW, and J-hG assisted in crafting the research questions and protocol. X-lL assisted with the statistical analysis. GH, Q-yW, and YY provided critical revisions that were important for intellectual content. All authors contributed toward selection of the studies and acquisition of data and contributed to the article and approved the submitted version.

## Funding

This research was supported by the Non-profit Central Research Institute Fund of Chinese Academy of Medical Sciences (No. 2020-PT320-001), National Natural Science Foundation of China (No. 81900040), and Liaoning Education Support Foundation (QN2019014).

## Conflict of Interest

The authors declare that the research was conducted in the absence of any commercial or financial relationships that could be construed as a potential conflict of interest.

## Publisher's Note

All claims expressed in this article are solely those of the authors and do not necessarily represent those of their affiliated organizations, or those of the publisher, the editors and the reviewers. Any product that may be evaluated in this article, or claim that may be made by its manufacturer, is not guaranteed or endorsed by the publisher.

## Glossary

COPD, chronic obstructive pulmonary disease; AECOPD, acute exacerbation of COPD; AE, acute exacerbation; TM, telemonitoring; SF, self-management; RCT, randomized controlled trial; FEV_1_, forced expiratory volume in 1 s; ER, emergency room; LOS, length of stay; HRQoL, health-related quality of life; SGRQ, St. George's Respiratory Questionnaire; EQ-5D, EuroQol five-dimension scale; EQ-VAS, EuroQol visual analog scale; HADS-A, Hospital Anxiety and Depression Scale-Anxiety; HADS-D, Hospital Anxiety and Depression Scale-Depression; 6MWT, 6-min walking test; SD, standard deviation; NR, not reported; BP, blood pressure; HR, heart rate; SPO_2_, pulse oxygen saturation; TEMP, temperature; PEF, peak expiratory flow; RR, respiratory rate; MD, mean difference; CI, confidence intervals; OR, odds ratio; RR, risk ratio.
