# Supplementary material for: Effectiveness of Telemonitoring for Reducing Exacerbation Occurrence in COPD Patients With Past Exacerbation History: A Systematic Review and Meta-Analysis
Source: Front Med (Lausanne). 2021 Sep 10;8:720019. doi: 10.3389/fmed.2021.720019 (PMC8460761; doi:10.3389/fmed.2021.720019)
Supplement: Supplementary file 2 [file Table_2.docx]

# Table S2: ‘Summary of findings’ table

**Patient or population**: AECOPD patients

**Setting**: At home

**Intervention**: Telemonitoring intervention

**Comparison**: Control group

| **Outcomes** | **№ of participants (studies) Follow up** | **Certainty of the evidence (GRADE)** | **Relative effect (95% CI)** | **Anticipated absolute effects** | |
| --- | --- | --- | --- | --- | --- |
|  |  |  |  | **Risk with Control group** | **Risk difference with Telemonitoring intervention** |
| ER visits  follow up: range 6 months to 12 months | 1099  (6 RCTs) | ⨁◯◯◯  VERY LOW  a,b,c | - | The mean ER visits was **1.37** | MD **0.7 lower**  (1.36 lower to  0.03 lower) |
| AE-related readmissions  follow up: range 6 months to 12 months | 1281  (7 RCTs) | ⨁◯◯◯  VERY LOW  a,b,c,d | **RR 0.74**  (0.60 to 0.92) | 459 per 1,000 | **119 fewer per**  **1,000**  (183 fewer to 37 fewer) |
| Range of AE-related readmissions follow up: range 6 months to 12 months | 1573  (9 RCTs) | ⨁⨁◯◯  LOW a,b,c | - | The median range of AE- related readmissions was **0.9** | MD **0.05 lower**  (0.14 lower to  0.05 higher) |
| All-cause readmissions  follow up: range 6 months to 12 months | 772  (4 RCTs) | ⨁◯◯◯  VERY LOW  a,b,c | **RR 0.92**  (0.78 to 1.08) | 455 per 1,000 | **36 fewer per**  **1,000**  (100 fewer to 36 more) |

***The risk in the intervention group** (and its 95% confidence interval) is based on the assumed risk in the comparison group and the **relative effect** of the intervention (and its 95% CI).

**CI,** Confidence interval; **MD,** Mean difference; **RR,** Risk ratio

# GRADE Working Group grades of evidence

**High certainty:** We are very confident that the true effect lies close to that of the estimate of the effect

**Moderate certainty:** We are moderately confident in the effect estimate: The true effect is likely to be close to the estimate of the effect, but there is a possibility that it is substantially different

**Low certainty:** Our confidence in the effect estimate is limited: The true effect may be substantially different from the estimate of the effect **Very low certainty:** We have very little confidence in the effect estimate: The true effect is likely to be substantially different from the estimate of effect

# Explanations

1. Risk of bias due to lack of blinding
2. Some results are contradictory to others
3. Downgraded due to small sample size and imprecision
4. have publication bias
