# Supplementary figures and images for: Effectiveness of Telemonitoring for Reducing Exacerbation Occurrence in COPD Patients With Past Exacerbation History: A Systematic Review and Meta-Analysis
Source: Front Med (Lausanne). 2021 Sep 10;8:720019. doi: 10.3389/fmed.2021.720019 (PMC8460761; doi:10.3389/fmed.2021.720019)

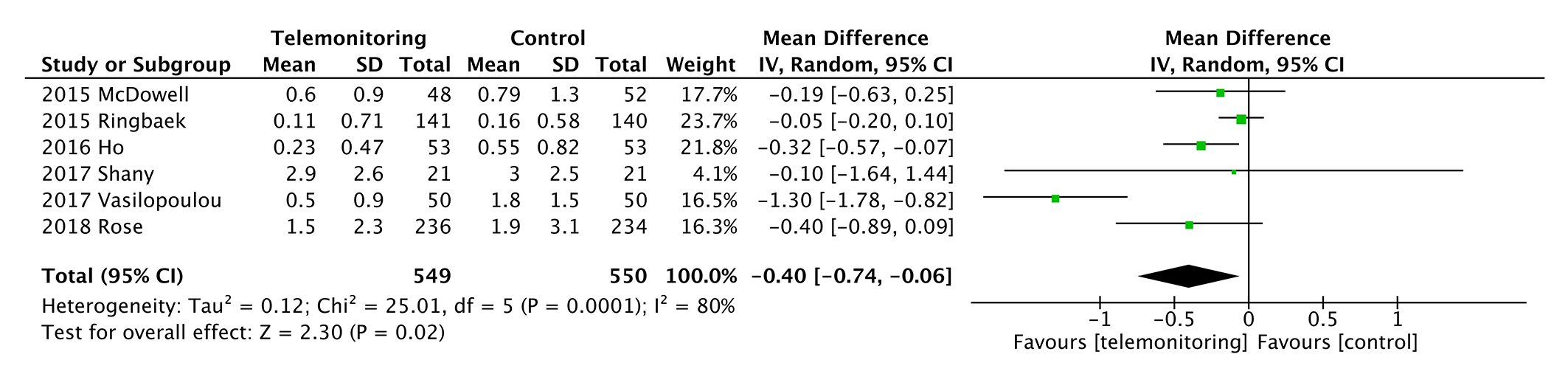

Supplement: Supplementary file 3 [file Image_1.TIF]
