# Supplementary material for: Effectiveness of Telemonitoring for Reducing Exacerbation Occurrence in COPD Patients With Past Exacerbation History: A Systematic Review and Meta-Analysis
Source: Front Med (Lausanne). 2021 Sep 10;8:720019. doi: 10.3389/fmed.2021.720019 (PMC8460761; doi:10.3389/fmed.2021.720019)
Supplement: Supplementary file 4 [file Data_Sheet_1.docx]

Supplementary Material

**Methods and Results**

**Methods**

**Search strategy**

**PubMed** N=245

((((((((((((((((((((mhealth[Title/Abstract]) OR remote[Title/Abstract]) OR tele[Title/Abstract]) OR telehealth[Title/Abstract]) OR ehealth[Title/Abstract]) OR (teleconsultation[Title/Abstract] OR teleconsultations[Title/Abstract])) OR telemonitoring[Title/Abstract]) OR telecare[Title/Abstract]) OR telehomecare[Title/Abstract]) OR telephone monitoring[Title/Abstract]) OR telepathology[Title/Abstract]) OR teleradiology[Title/Abstract]) OR (telecommunication[Title/Abstract] OR telecommunications[Title/Abstract])) OR telephone support[Title/Abstract]) OR teleconference[Title/Abstract]) OR call center[Title/Abstract])) OR (("Telemedicine"[Mesh]) OR "Remote Consultation"[Mesh]))) AND (("Pulmonary Disease, Chronic Obstructive"[Mesh]) OR COPD[Title/Abstract])) AND (("Disease Progression"[Mesh]) OR exacerbation[Title/Abstract] OR exacerbations[Title/Abstract])) AND (("Randomized Controlled Trial" [Publication Type] OR randomized[Title/Abstract] OR placebo[Title/Abstract]))

**Embase** N=68

Session Results

.......................................................

No. Query Results Results Date

#29. #20 AND #23 AND #27 AND #28 68 14 May 2020

#28. 'randomized controlled trial'/exp 600,988 14 May 2020

#27. #24 OR #25 OR #26 168,546 14 May 2020

#26. 'exacerbations':ab,ti 41,473 14 May 2020

#25. 'exacerbation':ab,ti 61,510 14 May 2020

#24. 'disease exacerbation'/exp 113,531 14 May 2020

#23. #21 OR #22 151,008 14 May 2020

#22. copd:ab,ti 85,975 14 May 2020

#21. 'chronic obstructive lung disease'/exp 133,950 14 May 2020

#20. #1 OR #2 OR #3 OR #4 OR #5 OR #6 OR #7 OR #8 OR 136,284 14 May 2020

#9 OR #10 OR #11 OR #12 OR #13 OR #14 OR #15 OR

#16 OR #17 OR #18 OR #19

#19. 'call center':ab,ti 794 14 May 2020

#18. 'teleconference':ab,ti 696 14 May 2020

#17. 'telephone support':ab,ti 994 14 May 2020

#16. telecommunications:ab,ti 2,065 14 May 2020

#15. telecommunication:ab,ti 2,196 14 May 2020

#14. teleradiology:ab,ti 1,095 14 May 2020

#13. telepathology:ab,ti 794 14 May 2020

#12. 'telephone monitoring':ab,ti 166 14 May 2020

#11. telehomecare:ab,ti 105 14 May 2020

#10. telecare:ab,ti 769 14 May 2020

#9. telemonitoring:ab,ti 2,170 14 May 2020

#8. teleconsultations:ab,ti 492 14 May 2020

#7. teleconsultation:ab,ti 1,019 14 May 2020

#6. ehealth:ab,ti 2,798 14 May 2020

#5. telehealth:ab,ti 4,878 14 May 2020

#4. tele:ab,ti 4,773 14 May 2020

#3. remote:ab,ti 88,833 14 May 2020

#2. mhealth:ab,ti 2,792 14 May 2020

#1. 'telemedicine'/exp OR 'teleconsultation'/exp 39,032 14 May 2020

**The Cochrane Library** N=192 (trails=192 review=3)

Date Run: 14/05/2020 15:58:34

Comment:

ID Search Hits

#1 MeSH descriptor: [Telemedicine] explode all trees 2380

#2 MeSH descriptor: [Remote Consultation] explode all trees 374

#3 (mhealth):ti,ab,kw 994

#4 (tele):ti,ab,kw 867

#5 (remote):ti,ab,kw 5041

#6 (telehealth):ti,ab,kw 1357

#7 (ehealth):ti,ab,kw 812

#8 (teleconsultation):ti,ab,kw 585

#9 (teleconsultations):ti,ab,kw 43

#10 (telemonitoring):ti,ab,kw 930

#11 (telecare):ti,ab,kw 177

#12 (telehomecare):ti,ab,kw 30

#13 (telephone monitoring):ti,ab,kw 1892

#14 (telepathology):ti,ab,kw 9

#15 (teleradiology):ti,ab,kw 28

#16 (telecommunication):ti,ab,kw 350

#17 (telecommunications):ti,ab,kw 175

#18 (telephone support):ti,ab,kw 4278

#19 (teleconference):ti,ab,kw 156

#20 (call center):ti,ab,kw 1179

#21 #1 or #2 or #3 or #4 or #5 or #6 or #7 or #8 or #9 or #10 or #11 or #12 or #13 or #14 or #15 or #16 or #17 or #18 or #19 or #20 16680

#22 MeSH descriptor: [Pulmonary Disease, Chronic Obstructive] explode all trees 5592

#23 (COPD):ti,ab,kw 15665

#24 #22 or #23 16741

#25 MeSH descriptor: [Disease Progression] explode all trees 7059

#26 (exacerbation or exacerbations):ti,ab,kw 15654

#27 #25 or #26 22156

#28 #21 and #24 and #27 195

**Study selection**

Two independent investigators (JW-L and YS) screened titles and abstracts to move out the irrelevant articles. Then the full-text of each remaining article was retrieved and evaluated against the inclusion and exclusion criteria. Any disagreements were resolved by consulting a third reviewer (GH) to decide eligibility.

**Data extraction**

In line with best practice, a standardized, piloted-tested form was developed by two independent reviewers to extract study details from the included studies. If consensus could not be reached, an additional reviewer reviewed data extraction and resolved conflicts. A narrative synthesis of the findings was then completed. In studies addressing several diagnoses, the results for patients with COPD will be separately evaluated. For the study with multiple or different control groups, we only pick out the pure control group without any telehealth intervention. In studies addressing several diagnoses, the results for patients with COPD will be separately evaluated.

**Quality assessment**

We presented overall risk of bias for each study with focus on random sequence generations, allocation concealment, blinding of participants, blinding of outcome assessment, attrition bias and reporting bias. Each domain was rated as “low risk”, “high risk” or “unclear risk” independently by two authors.

**Data synthesis and analysis**

The dichotomous variables included readmissions and mortality. And the continuous variables included ER visits, range of readmissions, length of stay, time to first hospital readmission, SGRQ, EQ-5D, EQ-VAS, HADS-A, HADS-D and 6MWT. Data were synthesized into forest plots. For dichotomous variables we calculated an odds ratio (OR) with 95% confidence intervals (95% CI) for individual studies and pooled results. For count data (i.e., where patients could have multiple events, such as the number of hospital readmissions), we calculated the rate ratio (RR; ratio of the incidence rate of events within a given time between the intervention and the control). For continuous variables we calculated mean difference (MD) and 95% confidence intervals for each study. If studies only reported the 95% confidence interval, then the standard deviations were calculated using the algorithms proposed by Cochrane Handbook: SD = (higher confidence limit -lower confidence limit)/3.92. When data are suspected to be non-normal, and reported minimum value, first quartile, median, third quartile, maximum value, range and sample size, we recommend the methods for estimating the sample mean and SD proposed by McGrath S, et al*. We quantified heterogeneity between studies using the I^2^ indicator with suggested thresholds for low (I^2^ = 0% to 49%), moderate (I^2^ = 50% to 74%), and high (I^2^ ≥ 75%) values. If I^2^ were greater than 50%, we used the random effect model; otherwise, we applied the fixed effect model. Pooled estimates set at P < 0.10 for heterogeneity testing.

**Grading the strength of evidence**

We planned to create a ‘Summary of findings’ table that includes key information concerning the quality of evidence, the magnitude of effect of the TM intervention and the sum of available data on the four outcomes.

• ER visits;

• Exacerbation-related readmissions;

• Range of exacerbation-related readmissions;

• All-cause readmissions;

We used the five GRADE considerations (study limitations, consistency of effect, imprecision, indirectness, and publication bias) to assess the quality of a body of evidence as related to studies that contributed data to the meta-analyses for prespecified outcomes. We employed methods and recommendations described in the Cochrane Handbook for Systematic Reviews of Interventions using GRADEpro software (Higgins 2011). We justified all decisions to downgrade or upgrade the quality of studies by using footnotes (Table S2).

**Results**

1. **LOS**

Six studies involving 1,037 patients were included in AE-related hospital days for exacerbation (Figure 5). There was a great reduction in the LOS between the TM group and the control group (*P* = 0.01, MD = -0.60, 95% CI [ -1.06, -0.13]). Slight heterogeneity was found (*P* = 0.31, I^2^ = 16%). Seven studies were included in hospital days for any cause (Figure 5), without statistically significant difference (*P* = 0.44, MD = -0.28, 95% CI [ -0.99, 0.42]). Heterogeneity was found in this comparison (*P* = 0.12, I^2^ = 41%).

**2. Mortality**

Figure 5 showed mortality in the TM groups and control groups. Eleven studies were included for mortality. The overall effect of the intervention on mortality was statistically significant in the included studies. (*P* = 0.01, OR = 0.71, 95% CI [0.54, 0.93]). Moderate heterogeneity was found (*P* = 0.08, I^2^ = 40%).

**3. Time to first hospital readmission**

Three studies were included for time to first hospital readmission (Figure 5). There was no statistically significant reduction between the TM group and the control group (*P* = 0.45, MD = -10.22, 95% CI [-36.76, 16.32]). High heterogeneity was found (*P* = 0.0001, I^2^ = 89%).

**4. Quality of life**

Different measurement tools for HRQoL were used in the various studies, included SGRQ, EQ-5D and EQ-VAS. Over 12 months of follow-up, the included studies showed lower mean SGRQ total scores (meaning better HRQoL) in the TM intervention compared with the control group. The MD of -3.72, (95% CI [-7.18, 0.26], *P* = 0.04), indicating better HRQoL in the intervention group compared to the control group, and heterogeneity was found (*P* = 0.05, I^2^ = 54%). In addition, ten studies encompassed the SGRQ baseline characteristics in the year prior to enrollment, there was no difference between groups(*P* = 0.44, MD = -0.65, 95% CI [-2.27, -0.98] and no heterogeneity was found (*P* = 0.46, I^2^ = 0%). In the EQ-5D component summary, which was only used by 2 studies, showed no improvement in quality of life (*P* = 0.90, MD = -0.03, 95% CI [-0.45, 0.40]), and no heterogeneity was found (*P* = 0.82, I^2^ = 0%). Likewise, in the EQ-VAS component summary, which was used in two studies, non-significant improvements in quality of life scores in both groups according to the VAS in the follow-up year (*P* = 0.84, MD = 4.54, 95 %CI [-39.37, 48.45]) and no statistical heterogeneity was observed between studies (*P* = 0.93, I^2^ = 0%). As shown in Table 3.

**5. Anxiety and depression**

The measurement tools for quality of life for analysis also included HADS-A and HADS-D. Two studies used the HADS-A, which did not show no significant difference between the TM group and the control group (*P* = 0.83, MD = 1.30, 95% CI [-10.43, 13.03]), and no heterogeneity was found (*P* = 0.92, I^2^ = 0%). Two studies used the HADS-D, which did not show a significant difference between the TM group and the control group either (*P* = 0.94, MD = 0.42, 95% CI [-10.26, 11.09]), and no heterogeneity was found (*P* = 0.95, I^2^ = 0%). See Figure 6.

**6. Exercise capacity**

We extracted data on change in exercise capacity. In Figure 6, only 2 studies contained 6MWT to determine the exercise capacity, which did not show a significant difference between the TM group and the control group (*P* = 0.33, MD = 35.63, 95% CI [ -35.63, 107.37]), and moderate heterogeneity was found (*P* = 0.12, I^2^ = 59%).

**References**

* McGrath S, Zhao X, Steele R, Thombs BD, Benedetti A; DEPRESsion Screening Data (DEPRESSD) Collaboration. Estimating the sample mean and standard deviation from commonly reported quantiles in meta-analysis [published online ahead of print, 2020 Jan 30]. Stat Methods Med Res. 2020;962280219889080. doi:10.1177/0962280219889080

**Supplementary Tables**

| **Table S1 Characteristics of included studies reported as mean (SD)** |
| --- |
| \| First author (Year)/  Region \| Study design/  Duration (months) \| Participants \| \| Intervention \| \| \| Control group \| Primary outcomes \| Results \| \| --- \| --- \| --- \| --- \| --- \| --- \| --- \| --- \| --- \| --- \| \| Characteristics (TM/control group) \| The history  of AE \| Type/  With SF or not \| Parameters monitoring \| Data trans-mission interval \| \| Casas[10]  (2006)  Spain, Belgium \| RCT  12 \| Sample size: 65/90 Age: 70 (9)/72 (9) Sex (Female%): 23/12 Lung function (using FEV1% predicted): 43 (20)/41 (15) \| A previous episode of exacerbation requiring hospitalization for 48 h. \| Integrated care including TM  Yes \| Biomedical parameters. \| NR \| Usual care \| 1. Hospital readmission. \| Integrated care effectively prevented hospitalizations for exacerbations. \| \| Antoniades[28]  (2012)  Australia \| RCT  12 \| Sample size: 22/22 Age: 70 (10)/68 (9) Sex (Female%): 55/55 Lung function (using FEV1% predicted): NR \| At least 1 hospital presentation in the last 12 months. \| Standard best practice care plus remote in-home TM  No \| BP, HR, SpO_2_, weight, TEMP, electrocardiogram, sputum color and volume, symptoms, medication usage. \| Everyday \| Standard best practice care \| 1. Hospital admissions;  2. Inpatient bed-days; 3. Quality of life. \| TM did not reduce healthcare utilization or improve quality of life. \| \| Jehn[29]  (2013)  Germany \| RCT  9 \| Sample size: 32/30 Age: 64.1 (10.9)/69.1 (9.2) Sex (Female%): 19/27 Lung function (using FEV1% predicted): 50.2 (15.0)/52.6 (17.5) \| At least 1 exacerbation during the previous year. \| TM intervention  No \| SpO_2_, PEF, FEV_1_, 6MWT. \| Five days per week \| Usual care \| 1. Clinical status and functional capacity \| TM reduced exacerbation frequency and health care utilization. \| \| Pinnock[14]  (2013)  UK \| RCT  12 \| Sample size: 128/128 Age: 69.4 (8.8)/68.4 (8.4) Sex (Female%): 59/51 Lung function (using FEV1% predicted): 44.0 (18.8)/40.0 (17.0) \| Had been admitted to hospital with an exacerbation of COPD in the previous year. \| TM integrated into existing clinical services  Yes \| SpO_2_, TEMP, symptom (dyspnea, fever, respiratory tract infection, cough, sputum purulence and volume, wheeze) \| Everyday \| Existing clinical services including SF advice \| 1. Time to first hospital admission with AECOPD. \| Not effective in postponing admissions and did not improve quality of life. \| \| Tabak[17]  (2014)  Netherlands \| RCT  9 \| Sample size: 15/14 Age: 64.1 (9.0)/62.8 (7.4) Sex (Female%): 50/50 Lung function (using FEV1% predicted): 50.0 (20.89)/36.0 (20.37) \| Three or more exacerbations or one hospitalization for respiratory problems in the 2 years preceding study entry. \| Telehealth program  Yes \| Major symptom (breathlessness, sputum, production, sputum color), minor symptom (wheeze, running nose, sore, throat, fever, cough). \| Everyday \| Usual care \| 1. Adherence to the online diary; 2. Adherence to the exercise scheme; 3. Satisfaction. \| With good satisfaction and with less frequent exacerbations. \| \| McDowell[30]  (2015)  Northern Ireland \| RCT  6 \| Sample size: 55/55 Age: 69.8 (7.1)/70.2 (7.4) Sex (Female%): 58.2/54.4 Lung function (using FEV1% predicted): 45.5 (13.7)/43.4 (11.3) \| At least 2 of: ER admissions, hospital admissions or emergency general practitioner contacts in the 12 months before the study. \| TM in addition to usual care  Yes \| HR, SpO_2_, BP and questions relating to symptoms (difficulty in breathing, cough, sputum, tiredness). \| Five continuous days per week \| Usual care \| 1. HRQoL. \| Effective in improving HRQoL and anxiety. No significant differences in ER visits, hospital admissions or exacerbations. \| \| Ringbaek[11]  (2015)  Denmark \| RCT  6 \| Sample size: 141/140 Age: 69.8 (9.0)/69.4 (10.1) Sex (Female%): 61/45 Lung function (using FEV1% predicted): 34.9 (13.3)/33.8 (12.0) \| Hospital admission due to COPD exacerbation within the previous 36 months. \| TM  No \| SpO_2_, weight, symptom (dyspnea, sputum, volume, purulence). \| Weekdays \| Usual care \| 1. The number of hospital admissions for exacerbation of COPD. \| TM did not reduce hospital admissions for exacerbated COPD. \| \| Cordova[31]  (2016)  USA \| RCT  24 \| Sample size: 34/33 Age: 64 (6)/63 (8) Sex (Female%): 50/73 Lung function (using FEV1% predicted): 31 (13)/32 (15) \| COPD hospitalization within the past year or current home oxygen use. \| Telemedicine-based home management program  No \| Breathlessness, sputum, PEF, TEMP, cough, wheeze, sore throat, nasal congestion. \| Everyday \| Usual medical care \| 1. The number of hospitalizations and deaths. \| TM facilitated early treatment of symptoms and improved lung function and functional status. \| \| Ho[12]  (2016)  Taiwan \| RCT  6 \| Sample size: 53/53 Age: 81.4 (7.8)/79.0 (9.6) Sex (Female%): 19/28 Lung function (using FEV1% predicted): 62 (23)/62 (21) \| COPD exacerbation as the main diagnosis. \| TM  No \| Weight, HR, SpO_2_, BP, TEMP, breathlessness, sputum. \| Everyday \| Usual care \| 1. Time to first readmission for COPD exacerbation. \| TM improved outcomes in terms of time to COPD-related re-admission, and average number of all-cause re-admissions and ER visits. \| \| Vianello[13]  (2016)  Italy \| RCT  12 \| Sample size: 230/104 Age: 75.96 (6.54)/76.48 (6.16) Sex (Female%): 29/27 Lung function (using FEV1% predicted):  41.90 (8.64)/41.87 (8.30) \| Adults at the time of discharge from hospital after an AE episode or attending the outpatient Pulmonary Clinics. \| TM  yes \| HR, SpO_2_. \| Weekdays \| Clinical care \| 1. HRQoL. \| TM did not significantly improve HRQL, not effective in reducing hospitalizations. \| \| Shany[5]  (2017)  Australia \| RCT  12 \| Sample size: 21/21 Age: 72.1 (7.5)/74.2 (9.0) Sex (Female%): 52/57 Lung function (using FEV1% predicted): 32.1 (16.0)/39.7 (13.2) \| At least 1 hospital admission for an exacerbation of COPD in the preceding year. \| Telehealth measurement and Respiratory Ambulatory Care Service-Plus.  No \| RR, HR, SpO_2_, BP, TEMP, weight, blood glucose, etc. \| Everyday \| Respiratory Ambulatory Care Service-Plus. \| 1. The change in the number of ER presentations;  2. Hospital admissions;  3. LOS; 4. When the primary diagnosis was an exacerbation of COPD during the year following randomization. \| Reduction in COPD-related admissions, ER presentations, and hospital bed days. \| \| Vasilopoulou[27]  (2017)  UK \| RCT  12 \| Sample size: 50/50 Age: 66.9 (9.6)/64.0 (8.0) Sex (Female%): 6/26 Lung function (using FEV1% predicted): 49.6 (21.9)/51.7 (21.0) \| A history of AEs of COPD 1 year prior to entering the study. \| Home-based maintenance tele-rehabilitation  Yes \| SpO_2_, HR, dyspnea and leg discomfort. \| Five days per week \| Usual care treatment \| 1. The rate of moderate to severe AE of COPD, hospitalizations and ED visits; 2. The rate of severe exacerbations (hospitalizations) and the rate of ED visits because of AE of COPD that did not require hospital admission. \| Equally effective in reducing the risk for AEs of COPD and hospitalizations and encounters a lower risk for ER visits. \| \| Kessler[32]  (2017)  France, Germany, Italy, Spain \| RCT  12 \| Sample size: 157/162 Age: 67.3 (8.9)/66.6 (9.6) Sex (Female%): 30.6/30.2 Lung function (using FEV1% predicted): 37.8 (12.4)/36.4 (12.3) \| At least 1 severe exacerbation in the previous year. \| A multicomponent home-based disease management intervention  Yes \| FEV_1_, SpO_2_, HR, O_2_ use, RR, symptom reporting \| Everyday \| Routine care \| 1. The number of unplanned all-cause hospitalization days. \| No significantly reduction in unplanned all-cause hospitalization days, but reduced acute care hospitalization days and mortality in severe COPD patients. \| \| Rose[33]  (2018)  Canada \| RCT  12 \| Sample size: 236/234 Age: 71.9 (9.2)/71 (9.7) Sex (Female%) 50/36 Lung function (using FEV1% predicted): 43(17.0)/45(17.8) \| ≥1 ER visit or hospital admission for COPD exacerbation in previous 12 months. \| A multi-component, case manager-led exacerbation prevention/management model  Yes \| Assessment of symptoms/symptom monitoring. \| Weekly \| Usual care \| 1. ER visits. \| No difference in ER visits, hospital admissions and other secondary outcomes. \| \| Soriano[9]  (2018)  Spain \| RCT  12 \| Sample size: 115/114 Age: 71.5 (8.0)/71.3 (8.9) Sex (Female%): 21.7/27.5 Lung function (using FEV1% predicted): 34.2 (9.1)/32.2 (8.8) \| Suffered 2 or more moderate or severe exacerbations in the previous year (with or without hospitalization). \| Telehealth  No \| BP, HR, SpO_2_, RR. \| Everyday \| Routine clinical practice \| 1. Change in the number of severe exacerbations (a hospital admission or a visit to the hospital emergency services). \| TM did not reduce COPD-related ER visits or hospital admissions \| \| Walker[16]  (2018)  Spain, UK, Slovenia, Estonia and Sweden \| RCT  9 \| Sample size: 154/158 Age: 71.0 (7.26)/71.0 (7.93) Sex (Female%): 34/34 Lung function (using FEV1% predicted): 49.4 (16.37)/50.4 (19.19) \| With a history of exacerbation in the previous year and at least 1 non-pulmonary co-morbidity. \| TM  No \| Lung mechanics, HR, SpO_2_, BP, TEMP. \| Everyday \| Usual care \| 1. Time to first hospitalization;  2. Change in EQ-5D utility index score. \| TM did not change time to first hospitalization and EQ-5D. \| \| Boer[6]  (2019)  Netherlands \| RCT  12 \| Sample size: 41/44 Age: 69.3 (8.8)/65.9 (8.9) Sex (Female%): 42/34 Lung function (using FEV1% predicted): 53.0 (21.5)/52.1 (19.8) \| Experienced 2 or more symptom-based exacerbations in the previous 12 months. \| A smart mobile health tool  Yes \| SpO_2_, TEMP, symptoms, physical limitations and emotions. \| Everyday \| A paper action plan \| 1. Exacerbation-free time, expressed as weeks without exacerbation. \| This study did not show beneficial effects on exacerbation-free time, health status, self-efficacy, SF behavior, and health care utilization. \|   Abbreviations: SD, Standard Deviation; SF, self-management; RCT, randomized controlled trial; NR, not reported; BP, blood pressure; HR, heart rate; SpO_2_, oxygen saturation; TEMP, temperature; PEF, peak expiratory flow; FEV_1_, forced expiratory volume in one second; RR, respiratory rate. See Table 1 legend for expansion of other abbreviations. There was no difference in demographic characteristics between the groups receiving TM intervention and the control groups regarding main clinical characteristics. All the patients included in the 17 articles had at least one exacerbation or one hospitalization for COPD prior to enrollment: patients of 12 articles had at least one AECOPD in previous 12 months, patients of others studies reported at least one AECOPD ranging from previous 48 hours to 36 months, 2 studies stating no specific time for AECOPD. The included studies were divided by intervention type: 8 studies used TM only, 9 studies used TM plus integrated care including SF and exercise. TM plus integrated care described in this systematic review was delivering home-based TM intervention with physical exercise, SF education and so on. Notably, 7 TM group added SF segment. All the studies monitored biological parameters (e.g., heart rate, respiratory rate, peripheral oxygen saturation, etc.) 5~7 days per week. There were 14 articles which control group only received usual care, the remain 3 articles received clinical care plus SF advice, Respiratory Ambulatory Care Service-Plus or a study-designed individualized plan, respectively.  **Table S3 Characteristics of TM’ technologies and devices**   \| Author (Year) \| Duration (month) \| Technologies \| Devices \| \| --- \| --- \| --- \| --- \| \| Casas[10](2006) \| 12 \| A standardized integrated care intervention, based on shared care arrangements among different levels of the system with support of information technologies: 1. a comprehensive assessment of the patient; 2. an educational programme on self-management of the disease administered; 3. agreement on an individually tailored care plan; 4. fourthly, accessibility of the specialized nurse to patients/carers and primary care professionals. Based on an information and communication technologies platform including a web-based call center. \| An information and communication technologies platform including a web-based call center. \| \| Antoniades[28] (2012) \| 12 \| The TM system comprised a laptop computer with digitally integrated blood pressure cuff and stethoscope, pulse oximeter, pneumotachograph for measurement of relaxed and forced spirometry, electrocardiogram touch plate, thermometer, and scales. The software provided a very simple user interface for guiding a patient through the series of measurements and was specifically designed for users with little or no computer experience. The system allowed patients to enter symptoms (visual analog scores measuring changes in overall health, dyspnea, sputum color and volume, symptoms of respiratory tract infection) and changes in medication usage (bronchodilators, inhaled and oral corticosteroids, and antibiotics). Data were uploaded daily to a central server via an Internet connection through the patient’s telephone line. \| Data were uploaded daily to a central server via an Internet connection through the patient’s telephone line. \| \| Jehn[29] (2013) \| 9 \| The TM intervention included 1. daily assessment of clinical status by means of the COPD Assessment Test (CAT); 2. daily lung function testing (spirometry); and 3. a weekly six-minute walk test measured by accelerometry. TM group patients completed all three testing modalities in their home-based environment. Following completion of the tests, patient data were transmitted via a mobile network directly to the study center. \| A mobile network. \| \| Pinnock[14] (2013) \| 12 \| Using a touch screen, TM participants recorded a daily questionnaire about symptoms and treatment use, and monitored oxygen saturation using linked instruments. Algorithms, based on the symptom score, generated alerts if readings were omitted or breached thresholds. \| A touch screen. \| \| Tabak[17] (2014) \| 9 \| The framework for the technology-supported care program, called the Condition Coach, was provided by previous work on self-management programs, home-exercise programs, changing activity behavior, and telemedicine interventions. The Condition Coach consists of four modules: teleconsultation, web-based exercising, self-management and activity coach. \| A technology-supported care program. \| \| McDowell[30] (2015) \| 6 \| 1. Telehealth monitor uploaded to record vital signs (blood pressure, heart rate, SaO_2_) and answers to questions relating to symptoms; 2. Patient monitored for five continuous days and report emailed to the local Community Respiratory Team Normal limits set; 3. Patient monitored for six months. \| A telehealth monitor. \| \| Ringbaek[11]  (2015) \| 6 \| The TM equipment comprised atablet computer with a web camera, a microphone, and measurement equipment(spirometer, pulse oximeter, and bathroom scale). Besides, patients reported changes in dyspnea, sputum color, volume, and purulence. The observations were transferred to a call center at each participant’s local hospital and automatically categorized and prioritized (i.e., green–yellow–red-coded). The call centers were open weekdays between 9 am and 3 pm and were staffed by a specially trained respiratory nurse. The nurse could confer the patients with a specialist in respiratory medicine at the hospital if values were alarming (a single measurement with red code or two consecutive measurements with yellow code); the patient was contacted by the respiratory nurse. \| A tablet cablet computer with a web camera, a microphone, and measurement equipment. \| \| Cordova[31]  (2016) \| 24 \| All patients were instructed to report their symptoms daily using the electronic diary. The electronic diary had eight screens of questions that took 2–3 min to complete. Patients obtained three peak flow readings using a handheld disposable peak flow meter. The patient responded to the prompts on each of the screens regarding his or her symptoms that day and entered the best peak flow meter reading. Once all data were entered, the electronic diary was placed into its cradle, and the daily symptom report was transmitted by telephone line to a central database. The data submitted by the patient were processed by the algorithm. \| An electronic diary was placed into its cradle, and the daily symptom report was transmitted by telephone line to a central database. \| \| Ho[12] (2016) \| 6 \| The patients were instructed to report their symptoms using the electronic diary on the website each day for two months after discharge. The diary consisted of eight questions involving disease-related symptoms, vital signs and weight, and took about two min to complete. The submitted data were processed according to the predefined algorithm, which was established by the study team in a round table conference. The indicators chosen and the scores assigned to each criterion were determined taking into account COPD symptomatology and common physiological responses to illness and by consensus. Once a warning was generated, the study nurses and attending pulmonologists received a notification to respond to the situation. The study pulmonologist would assess the patient’s data in light of the patient history, with the option of contacting and evaluating the patient by phone as clinically indicated. Based on the best clinical judgment, the patient could be referred to the clinic or ER. \| An electronic diary on the website. \| \| Vianello[13]  (2016) \| 12 \| Patients in the intervention group were provided a TM system consisting of a finger pulse-oxymeter and a gateway device for data transmission over a telephone line to a central data management unit located at the Veneto Regional e-Health Center. \| A telephone line to a central data management unit. \| \| Shany[5] (2017) \| 12 \| Patients were instructed to record their measurements on the telehealth units once daily, at any time. Every night, the collated information was automatically sent via the Internet (over a phone line or broadband) to a secure central server. Patients could also take additional measurements that would be immediately sent to the server. All collected information was presented to the hospital-based Respiratory Ambulatory Care Service-Plus staff and the authorized researchers of this project on a similarly protected website. The measurements were presented as a webpage of the current day’s measurements as well as graphs of the measurements over time. An alert system was available on the website to flag patients with measurements that breached predefined thresholds. \| The Internet (over a phone line or broadband). \| \| Vasilopoulou[27] (2017) \| 12 \| The home-based maintenance tele-rehabilitation programme consisted of 144 sessions performed over 12 months. The programme included the following components: 1. Individualized action plan; 2. physical exercise sessions to remote monitoring; 3. access to the call center 5 days per week, 10 h per day; 4. psychological support; and 5. dietary and self-management advice via scheduled weekly contacts with a physiotherapist, an exercise scientist, a dietician and a physician through telephone or a video conference. \| Telephone or a video conference. \| \| Kessler[32] (2017) \| 12 \| Home monitoring consisted of the transmission of health status information by patients using a telephone-based questionnaire at least once per week, and each day they began to experience symptom worsening. An e-health telephone/web platform allowed timely patient follow-up by case managers for early detection of potential exacerbations and symptom worsening. Information was transmitted to the hospital physician via the web platform to coordinate healthcare and early treatment when necessary. Physicians made all decisions regarding medication to reduce the risk of inappropriate medication. \| An e-health telephone/web platform. \| \| Rose[33] (2018) \| 12 \| 1. Case-manager delivered 40-min standardized education session based on Living Well with COPD on study enrolment; 2. Individualized care and action plans for COPD exacerbation recognition, self-management and management of comorbidities; 3. Case manager-initiated telephone consultations (12 weekly, and monthly for the subsequent 9 months; 21 sessions) comprising standardized reinforcement/motivational interviewing focusing on health behaviors; action plan teach-back sessions; assessment of symptoms/symptom monitoring, problems and problem solving strategies; 4. Ongoing case manager communication with family physicians and with hospital specialists including respirologists; and 5. Priority access to ambulatory outpatient clinics. Exacerbation management prescriptions were provided with the action plan either directly to the participant or to their pharmacy. \| Telephone. \| \| Soriano[9] (2018) \| 12 \| This was performed by healthcare personnel who installed monitoring equipment, trained the patient or carer on its use. The equipment given to each patient comprised a pulse oximeter, a blood pressure gauge, a spirometer and a respiratory rate and oxygen therapy compliance monitor VisionOx® connected to the oxygen feed from their main oxygen source. All were connected to a free 3G modem (2Net Hub) which uploaded readings to secure servers. Blood pressure, oxygen saturation, heart rate and spirometry were actively measured by the patient at home as per instructions whilst respiratory rate (and oxygen adherence) data was passively collected by the Visionox® device. The patient took measurements at the same time daily, at rest and after having taken their prescribed medication and with the oxygen therapy. \| Monitoring equipment connected to a free 3G modem (2Net Hub). \| \| Walker[16] (2018) \| 9 \| The intervention group used the CHROMED monitoring platform for nine months at approximately the same time each day. The plat form comprised a device which measured within-breath respiratory mechanical impedance using FOT (forced oscillation technique), a touch-screen computer and a mobile modem. FOT measurements were cleaned automatically from artefacts using previously published filtering rules and automatically sent to the study server. An algorithm generated respiratory alerts if a trend of worsening was detected in at least one of the following FOT parameters measured at 5Hz. \| CHROMED monitoring platform via a touch-screen computer and a mobile modem. \| \| Boer[6] (2019) \| 12 \| Participants in the intervention group were instructed to visit the nurse within 2 weeks after allocation for instructions on the use of the mHealth tool. The tool consisted of a mobile phone(provided by the research team), a pulse oximeter, a spirometer, and a forehead thermometer. Patients answered 12 yes-or-no questions concerning changes in symptoms, physical limitations, and emotions using the touch screen on the mobile phone complemented by measurements with the pulse oximeter, spirometer, and forehead thermometer. \| A mHealth tool using the touch screen on the mobile phone. \|   **Figure Legend**  **Figure S1: Meta-analysis. ER visits.**In the study of Vasilopoulou, 2017, when we changed the hospital-based rehabilitation group instead of usual group as a comparator. The heterogeneity didn’t have changed distinctively (from 90% to 80%, *P* = 0.02) |
